# Health Information Literacy and Competencies of Information Age Students: Results From the Interactive Online Research Readiness Self-Assessment (RRSA)

**DOI:** 10.2196/jmir.8.2.e6

**Published:** 2006-04-21

**Authors:** Lana Ivanitskaya, Irene O’Boyle, Anne Marie Casey

**Affiliations:** ^1^Central Michigan UniversityMt. Pleasant, MIUSA

**Keywords:** Health information, electronic health information, evaluation of electronic resources, electronics, telecommunications, consumer health information, patient education, educational status, computer network

## Abstract

**Background:**

In an era of easy access to information, university students who will soon enter health professions need to develop their information competencies. The Research Readiness Self-Assessment (RRSA) is based on the Information Literacy Competency Standards for Higher Education, and it measures proficiency in obtaining health information, evaluating the quality of health information, and understanding plagiarism.

**Objective:**

This study aimed to measure the proficiency of college-age health information consumers in finding and evaluating electronic health information; to assess their ability to discriminate between peer-reviewed scholarly resources and opinion pieces or sales pitches; and to examine the extent to which they are aware of their level of health information competency.

**Methods:**

An interactive 56-item online assessment, the Research Readiness Self-Assessment (RRSA), was used to measure the health information competencies of university students. We invited 400 students to take part in the study, and 308 participated, giving a response rate of 77%. The RRSA included multiple-choice questions and problem-based exercises. Declarative and procedural knowledge were assessed in three domains: finding health information, evaluating health information, and understanding plagiarism. Actual performance was contrasted with self-reported skill level. Upon answering all questions, students received a results page that summarized their numerical results and displayed individually tailored feedback composed by an experienced librarian.

**Results:**

Even though most students (89%) understood that a one-keyword search is likely to return too many documents, few students were able to narrow a search by using multiple search categories simultaneously or by employing Boolean operators. In addition, nearly half of the respondents had trouble discriminating between primary and secondary sources of information as well as between references to journal articles and other published documents. When presented with questionable websites on nonexistent nutritional supplements, only 50% of respondents were able to correctly identify the website with the most trustworthy features. Less than a quarter of study participants reached the correct conclusion that none of the websites made a good case for taking the nutritional supplements. Up to 45% of students were unsure if they needed to provide references for ideas expressed in paraphrased sentences or sentences whose structure they modified. Most respondents (84%) believed that their research skills were good, very good, or excellent. Students’ self-perceptions of skill tended to increase with increasing level of education. Self-reported skills were weakly correlated with actual skill level, operationalized as the overall RRSA score (Cronbach alpha = .78 for 56 RRSA items).

**Conclusions:**

While the majority of students think that their research skills are good or excellent, many of them are unable to conduct advanced information searches, judge the trustworthiness of health-related websites and articles, and differentiate between various information sources. Students’ self-reports may not be an accurate predictor of their actual health information competencies.

## Introduction

### Background and Purpose of the Study

As society moves toward evidence-based medicine [1], health providers, health educators, and health care consumers must acquire not only basic health information literacy skills but also more advanced competencies [2]. These competencies include evaluation of the quality of health information resources, obtaining health information documents on narrow topics by conducting advanced searches, judging the trustworthiness of health information sources, and understanding the advantages and disadvantages of different media. The last point is of special concern because many individuals have come to rely on the Internet as a main source of health information. This research addresses the Healthy People 2010 Objective 11-2, currently worded as “to improve the health literacy of persons with inadequate or marginal literacy skills,” but which may be expanded to the entire US population instead of only to those with marginal or inadequate literacy skills [3]. In addition, it aims at providing needs assessment information that may aid in accomplishing Objective 11-3, which is related to increasing the proportion of health communication activities that include research and evaluation, and Objective 11-4, set to increase the proportion of health-related websites that disclose information that can be used to assess the quality of the sites.

Recent reports suggest that over 55% of Americans with Internet access seek health information online [4]. One of the most common complaints about online health information searches is the amount of time required to process the documents that are found [5], but this observation is likely to be related to the general nature of the searches conducted—few information consumers use advanced search features, precisely specify their keywords, or limit their searches in some other way. While Internet search engines help identify a very large number of health-related documents, their use calls for advanced competencies that not all information consumers may possess. For example, the vast majority of documents found on the Internet have not passed a rigorous peer-review process. The ability to conduct one’s own review is clearly an advanced skill. Arguably, health information consumers will be at a greater risk of making health decisions on the basis of noncredible information if they conduct a Google search as opposed to a search in a scholarly library database. This risk will be particularly high for individuals with poor health information competencies. Research comparing clinical evidence to Internet information reveals numerous examples of erroneous and potentially harmful information on such popular topics as cancer rates, smoking cessation methods, and fever management in children [6-8].

Internet users may tend to underestimate the effort and competence required for obtaining trustworthy health information. A decade ago, communication researchers who compared print and television media described this paradox:

[Individuals] have learned that print materials, so highly prized in school and elsewhere, are indeed more difficult to process, whereas TV can be processed for pleasure without much effort. However, this argument pertains only to the minimum effort needed for the satisfactory processing of materials; it says nothing about the amount of additional effort one *could* expend in processing televised material if one aimed at a deeper understanding of it[9].

Although the Internet provides access to a vast number of documents on health-related topics, it is hard to build evidence-based knowledge about a health issue if one cannot determine the credibility of websites and the trustworthiness of the online documents. The *minimum* effort required for identifying millions of websites on a particular health topic is in sharp contrast with the *average* effort required to sift through the gigabytes of information in order to sort out the most credible documents, or at least those that appear as such.

Higher education institutions in the United States provide access to an unprecedented quantity of digital information via library archives, licensed online databases, and the public-access Internet. To differentiate between publicly accessible Web documents and password-protected scholarly databases, which can be accessed by paid members via the Web, we refer to the former as the “the public-access Internet.”

Our study explores three basic questions: How proficient are university students at finding and evaluating health-related information? How well do they understand the difference between peer-reviewed scholarly resources and opinion pieces or sales pitches? How aware are they of their own level of health information competencies? The main goal of this project was to identify approaches to building Information Age competencies of young health consumers, specifically a cohort of 18- to 23-year-old students enrolled in higher education programs.

### Literature Review: Health Information and the Internet

In accordance with the Healthy People 2010 health communication objective [3], public health professionals attempt to assist consumers seeking health information via the Internet, for instance, by reinforcing the need for quality standards and widespread criteria for evaluating health information [10-14]. Cline and Haynes [10] note that, while critics are fast to question the quality of online health information, limited empirical research on this topic does not allow any broad conclusions to be drawn. In a study published the same year, Eysenbach and colleagues [15] reported that Internet coverage of health information was often inconsistent, although the accuracy was generally good, and that search engines and simple search terms did not provide efficient access to health information. Crespo [16] reviewed several studies on online health information seekers and concluded that most users seemed to focus on finding information quickly rather than on evaluating the information found. Similarly, Eysenbach and Kohler [17] found that individuals explored only the first few links obtained from a search using a general search engine. Although some Internet users attempted to assess the credibility of sites by, for example, examining their source and professional designs, many people did not read the “about us” sections of websites, learn about the authors or owners of the sites, or review disclaimers and disclosure statements. Very few Internet users later remembered from which websites they retrieved information or who stood behind the sites [17].

Thus, abundance of health information does not always translate into informed choices. Hibbard and Peters [18] suggest that three factors should be considered in selecting information presentation strategies: (1) the complexity and amount of information; (2) the nature of the choice—degree to which there is a right or best option; and (3) the experience, motivation, and skills of users. The third point, deficient information skills, may prevent members of the public from recognizing that key information is missing, from understanding the difference between biased and unbiased information, from distinguishing evidence-based claims, and from interpreting the information intended for health professionals [10]. Researchers, having observed individuals who, on average, spent about one-half hour looking for health information, concluded that information consumers should have at least a tenth grade reading level to process Web materials. Many websites presented to the participants of this study contained material at a college level [15].

Online health care is having a growing cultural impact, affecting the practitioner-patient relationship and opening up the possibility of new roles for social workers and educators in the provision of health services [19]. The increasing use of the Internet draws scientists’ attention to modeling individual behavior, contributing to the development and refinement of individual health theories and models, such as the Theory of Planned Behavior, The Health Belief Model, and The Transtheoretical Model [20]. The theoretical framework for this study is largely based on the information processing theories and concepts discussed below.

Schneider and Shiffrin [21] distinguish two qualitatively different modes: (1) conscious, intentional processing of information that is capacity limited (*controlled processing*), and (2) quick and efficient *automatic processing* of information that has greater capacity, for example, when several tasks can be done at the same time. Automaticity requires less attentional resources than controlled processing, and it is developed through extensive practice under the condition of consistent stimuli and response requirements. When surfing the Internet, for example, health information consumers limit their exposure to inconsistent conditions—they tend to use the same search engines and the same searching methods, such as entering keywords into the nonadvanced search window. The assessment of health information competencies in this study incorporates tasks that call for automatic processing and tasks where stimuli and response requirements of the task are inconsistent with most health information consumers’ information search practices.

We also draw upon Anderson’s ACT theory [22,23], which explains skill acquisition. It incorporates research on automaticity and explains the development of cognitive skills important for processing digitized health information from a variety of electronic sources [24]. According to Anderson [22,23], skill development has three stages: (1) the declarative knowledge stage, when knowledge of facts is built, such as facts about reputable sources of health information and general procedures for obtaining information; (2) the knowledge compilation stage, which is characterized by proceduralization and composition; and (3) the procedural stage. To illustrate the second stage, consider a health information consumer who follows a set sequence of specific steps to search for a health-related terms (*proceduralization*) and reapplies this sequence until sufficient information on a health topic is found (*composition*). Once at the knowledge compilation stage, a consumer can perform an information search task at a higher speed and with fewer errors than at the declarative knowledge stage. High speed and low error rate are both important markers of skilled performance. However, a disadvantage of knowledge compilation is the rigidity of behavior, when individuals find it increasingly difficult to attend to intermediate feedback (e.g., step-related results) and engage in strategy modification (e.g., by adopting a search strategy that produces a greater number of trustworthy health information resources) [24]. Declarative and procedural knowledge are discussed in greater depth in the Methods section.

### An Interdisciplinary Research Partnership

Our research originated from the collaboration of a psychologist, a health educator, and a librarian who set out to understand and improve health information competencies of the Information Age generation. The collaboration enhances our research in several ways. The psychologist contributes expertise in the area of psychometrics and test design, whereas the health educator contributes knowledge of health consumers’ behavior and intervention designs. The librarian contributes expertise in training and enhancing patrons’ health information–seeking skills [25], as well as knowledge about gateways to authoritative consumer health information, for example, Medline Plus [26,27]. Linnan and colleagues [28] believe that library/public health partnerships are capable of increasing information access, the quality of available health information, and the technological expertise of all community members. Neighborhood libraries often serve the online health information needs of consumers who may not have Internet access at home, such as the elderly, ethnic groups, and low-income and undereducated populations [29,30], whereas university libraries also serve as gateways to scholarly health materials that are not available on the public-access Internet. In addition to public-access health resources available online, this research focuses on scholarly health resources in academic libraries and their use by students who are training to become health professionals.

## Methods

### Participants

A sample of 400 college-age students was selected because this cohort is the first Information Age generation that has been exposed, for up to one-half of their lives, to the Internet. Students enrolled in three courses in the College of Health Sciences at a Midwestern university were invited to participate in the study. The first class was a high-enrollment introductory course on the determinants of health. Although only undergraduate students (n = 354) participated in this course, they represented all levels of undergraduates—freshman (59%), sophomores (22%), juniors (9%), and seniors (10%). The second class was an advanced course in health administration in which both undergraduate (n = 19) and graduate students (n = 3) were enrolled. The third class was a mid-level health education course (n = 25) for undergraduate students. All students enrolled in the advanced health administration course and the mid-level health education course were majoring in health professions. About one third of the introductory course students with declared majors were majoring in a health-related discipline, and 31% of students had not made up their minds about a major field of study.

Introductory course students completed the assessment for extra credit, while others did it to learn more about their own skills. The instructors emphasized that the purpose of the assessment was to help students become competent consumers of health-related information.

### Measures

#### Health Information Competencies

Ivanitskaya and Casey developed the Research Readiness Self-Assessment (RRSA) to measure basic research skills based on the Information Literacy Competency Standards for Higher Education developed by the Association of College and Research Libraries [2,31]. The RRSA designers’ original intent was to measure information competencies, both general and discipline specific, of students attending colleges and universities. A health information version of the RRSA is discussed in this paper; it was created to specifically evaluate health information competencies. Competencies are knowledge/skills sets essential for accomplishing a goal, in this case, finding quality information on a specific health topic. The RRSA measures competencies linked to such college-age health information consumer behaviors as determining possible sources of health information, conducting health information searches, evaluating the quality of documents found, and using those documents appropriately. One of the relevant competencies is knowledge of plagiarism because it can be applied to properly recognize ideas contributed by others and to evaluate health-related documents. The RRSA designers aimed at measuring *foundational* competencies that are (1) transferable to other knowledge domains (e.g., social sciences in addition to health sciences); (2) applicable to a large number of health information consumers; (3) consistent with typical behaviors or experiences of health information consumers who seek information from electronic sources; and (4) that capture the nature and spirit of critical thinking, life-long learning, and advances in information technology. It is important to note that the RRSA instrument does not measure higher order skills that characterize experienced researchers, such as the design of clinical trials [31]. The word *research* in the assessment’s title matches the language commonly used by the lay population, as in “going to Google to research a health topic,” which is indicative of such behaviors as searching, judging, and making decisions.

The RRSA contains the following items: (1) multiple choice or true/false questions that measure declarative knowledge; (2) interactive, problem-based exercises that measure procedural knowledge; (3) demographic questions; and (4) a question that asks for a self-report about the level of the respondent’s research skills [31].

Declarative knowledge, defined as knowledge of facts or verbal knowledge, is a precursor to higher-order learning, which is needed, for example, to complete a sequence of steps to critically analyze a website or to employ elegant information search strategies [32]. Declarative knowledge questions in the RRSA measure knowledge of plagiarism, health information sources, and research-related terminology. For example, the following item is used to measure knowledge of research-related terminology:

A journal article abstract is…

1. an annotated list of references used in the article
2. a summary of the article’s content
3. a summary of other research on this topic
4. a note or paragraph about the authors of the article
5. a glossary of abstract concepts included in the researcher’s model

Compared to declarative knowledge, procedural knowledge is related to skills and problem solving. Essential for reproduction of learned behaviors, procedural knowledge is defined as knowledge of the process used to complete a task (e.g., how an information search process can be sequenced, organized, or controlled) [32]. In the RRSA, problem-based interactive exercises are used to measure procedural knowledge. Procedural knowledge questions include links to websites, library catalogs, and interactive search modules designed specifically for the RRSA. Students demonstrate their database navigation skills by setting up basic and advanced searches. For example, the following item is used to measure skill in conducting a search using Boolean operators (*and, or, not*):

You are interested in gathering information about work stress but are not interested in its medical side effects. Set up a document search in a separate window using the following keywords: *stress* *medical*. Click here to begin your search [a hyperlink to an interactive online module similar to searches in health-related library databases, such as Medline, with text fields for entering key words and a choice of Boolean operators]. Report the number of documents you found: a) 255; b) 555; c) 700; d) 1164; e) 55164.

In addition, students evaluate the quality of research publications, make judgments about website trustworthiness, and detect plagiarism. For example, the following item is used to measure evaluation of the trustworthiness of websites:

You are looking for information on various nutritional supplements. You found three websites. Click on the links below to examine each site and to evaluate its content. Which of these websites is the most trustworthy? a) cognitogenic aids [a hyperlink]; b) dormitogenic aids [a hyperlink]; c) vescorogenic (gustatogenic) aids [a hyperlink].

#### Instrument Piloting and Validation

To pilot test an earlier version of the RRSA instrument and to gather initial evidence about its validity and reliability, we administered a 60-item assessment to undergraduates (n = 100) and doctoral students (n = 45), as well as professional librarians (n = 5) and health professionals (n = 3). The feedback from librarians and health professionals offered preliminary evidence in support of the instrument’s face validity and content validity. Specifically, the librarians confirmed that the items included in the RRSA assessment conformed to the Information Literacy Competency Standards and addressed knowledge and skills important to health information consumers. The wording of several items, both stems and response options, was revised based on librarians’ recommendations. In addition, the librarians completed the assessment themselves. Their scores were then compared to the scores of students at two academic levels, undergraduate and doctoral. The results indicated that individuals with greater training and experience in managing digital health information performed better than individuals with less experience. Undergraduate students’ overall scores were the lowest (about 66% correct responses), followed by doctoral students’ scores (73%) and librarians’ scores (95%). These results offer preliminary evidence of the assessment’s criterion-related validity. The pilot test indicated an acceptable internal consistency value (Cronbach alpha > .70), although it could be improved (approach .80) if four items were removed. Therefore, four RRSA items that reduced the overall internal consistency were deleted.

The revised assessment contains 56 items, including 16 multiple-choice questions and 40 true/false questions grouped under 7 stems (Multimedia Appendix 1). For example, knowledge of information sources is measured by a stem that states, “Which of these citations are to journal articles?” The participants then check all that apply from the list of 6 true/false items (3 references to journal articles, 1 book reference, and 1 book chapter reference). Items are scored as +1 if the answer is a correct positive or a correct negative and +0 if the answer is a false positive or a false negative. Further description of the development of the stimulus materials used in website evaluation appears in the Results section, under Proficiency in Evaluating Health Information.

The RRSA assessment was designed to be useable by more than one institution. Its content can be adapted to the needs of various educational programs. Specifically, instructions to participants, the text of individual questions, detailed feedback, links to additional resources, and disclaimers (e.g., about participants’ rights and how the information they provide will be used) can be revised, without help from programmers, using the password-protected online control panel. This has been done by three US universities and one Canadian university that adopted the RRSA for use in their academic programs. For example, all four institutions revised search questions to enable their students to search for documents in their own university’s library catalog. The original RRSA designers provide coaching and training in order to ensure that the changes made to the RRSA do not have a negative impact on its reliability and validity. Ongoing validation studies provide a quality control mechanism and allow the testing of new or revised questions suggested by the partner institutions. The administration of the RRSA to partner institutions is supported through grants, partner donations, and volunteer efforts by the RRSA design team members.

### Other Measures

We asked the study participants to share information about their age, gender, and education. Self-reported level of research skills was measured with a single item, “How do you rate your research skills?” with six response options ranging from 1 (nonexistent) to 6 (excellent).

### Procedures

The RRSA instrument was administered online. Each student was issued a unique pass to access RRSA questions. The students had the option of submitting an incomplete survey and then returning to it at a later time to finish the remaining questions. This feature promoted better information processing and relieved the students from the need to rush and finish the entire assessment on their first attempt. The average estimated RRSA completion time was 26 minutes. Upon answering all questions, the students received an individualized results page that summarized their performance in different areas by providing a score, a maximum possible score, and percent attained. In addition to the numerical RRSA results, the Web page displayed individually tailored feedback composed by an experienced librarian. The Web page was programmed to compare, within each performance category, each individual student’s performance to the performance of a norm group. In accordance with the student’s competency level, the feedback provided suggestions for skill improvement and an explanation of factors that may have contributed to low, average, or high performance in each area. Finally, students who completed the RRSA were given the option to request additional materials for remedial learning, such as an explanation of the difference between scholarly and nonscholarly resources. The links to these additional materials were delivered to students via email.

### Data Analyses

Descriptive statistics were used to examine respondents’ performance in four areas—searching for health-related information, understanding plagiarism, evaluating health information, and self-reported skill level. To examine the relationship between self-reported skill level and actual performance, we computed composite scores. A composite overall score, which is indicative of the health information competency level, was created by adding points for 56 items, which were either true/false or multiple choice. Composite score calculations were preceded by an internal consistency reliability analysis that determined the appropriateness of combining responses from multiple items. We used a Spearman correlation to assess the relationship between the actual skill level (overall score) and self-reported skill level. A multiple regression analysis was used to examine the relationship between actual performance and perceived skill while holding the amount of education (number of credit hours earned) constant.

## Results

Our research questions were the following: How proficient are university students at searching for and evaluating health-related information? How well do they understand the difference between peer-reviewed scholarly resources and opinion pieces or sales pitches? How aware are they of their own level of health information competencies? The results for each question are presented below, preceded by a sample description.

### Respondent Characteristics

The participation rate was 77%. Nonrespondents (n = 92) differed from respondents (n = 308) in terms of their academic level (*t*_400_ = 2.29, *P* = .02). Freshmen were slightly more likely not to participate in the RRSA than seniors; the participant group included 7% less freshmen and 10% more seniors than the nonparticipant group. Most respondents were female (77%) and between 18 and 23 years of age (95%). The vast majority of respondents (98%) did not have a bachelor’s degree, and the remaining students were working toward their master’s degrees. Because we administered the RRSA to students in health professions courses, over one third of respondents were majoring in health sciences. Common majors were athletic training and sports medicine, health administration, physical education, pre-physical therapy, and public health promotion. On average, the undergraduates who participated in the study had completed 40 or fewer semester credit hours of university coursework. A quarter of respondents reported earning over 71 credit hours.

### Proficiency in Searching for Health Information

Table 1 summarizes performance in searching for health information. The data indicate that most students recognize common health journal titles and can perform a basic search in a library catalog, for example, by entering an exact book title into the title search. Few students, however, can perform an advanced search for a book when they know the book’s author (with a very common last name), general topic, and publication date. We call this search advanced because imprecise book specifications make it hard to find the book without performing a search that takes into account all or nearly all of the available information.

The data also show that two thirds of study participants are unable to understand or apply Boolean operators, such as *and*, *or*, and *not*. Boolean operators are used in most search engines, including those used for navigating the Internet (Google or Yahoo), library databases with scholarly journal articles, and library catalogs. Even though most students (89%) understand that a one-keyword search is likely to return too many documents, few are able to narrow a search by using multiple search categories simultaneously or by employing the Boolean operators. In addition, nearly half of the respondents have trouble discriminating between primary and secondary sources of information, as well as between references to journal articles or other published documents, such as books or book chapters.

### Proficiency in Evaluating Health Information

One of the most important markers of a competent health information consumer—critical judgment of information—is assessed in two ways: (1) the first set of questions calls for a review of three full-text articles from journals, and (2) the second set of questions calls for a comparison of three health-related websites.

The three journal articles are on the topic of job satisfaction, a topic relevant to any profession, and come from a full-text library research database. They include a rigorous empirical study, a case study, and an opinion article. Only the empirical study has a bibliography and an explicit statement about the author’s affiliation. The opinion article, clearly the least authoritative source, makes no mention of the author’s affiliation. As shown in Table 1, most respondents can determine the article publication date; it appears at the top of a full-text article. Many respondents can also identify an opinion article. Fewer respondents know how to determine if an article includes a research review and are able to check for the author’s affiliation.

The three Web pages about nutritional supplements are realistic looking interactive screens that appear to be live websites. The content of these mock websites, developed specifically for the RRSA, includes graphics, hyperlinks, and text about nonexistent classes of nutritional supplements—cognitogenics, dormitogenics, and gustatogenics. Each website is dedicated to one class of supplement and explains its purpose (e.g., cognitogenics help people with learning disabilities), prevalence (e.g., “gustatogenic aids have been available in Germany and Canada for over five years”), and safety. Even though the descriptions of nutritional supplements were fictitious, all three websites accurately stated that the US Food and Drug Administration did not evaluate the safety or benefits of these nutritional supplements.

**Table 1:** Searching and evaluating health information: performance on select measures (n = 308)

	**Respondents With Correct Answers**	
	**n**	**%**
**Searching for Health Information**		
Knowledge of a scholarly source, *Journal of American Medical Association* (7)	293	95
Demonstration of a skill in locating a book in a university library catalogue based on its exact title (16)	286	93
Understanding that a one-keyword generic search may return too many documents—an overwhelmingly large number of resources on a variety of topics (4)	275	89
Use of a proper research strategy—thinking about a broad topic to identify a sub-area of interest (2)	268	87
Ability to detect a journal citation that is incomplete—lacks a year of publication (17)	241	78
Understanding of a term “article abstract”—a summary of the article’s content (8)	234	76
Knowledge that a journal is a source of scholarly (analytical) information on a narrowly specialized topic (6)	214	70
Understanding of a term “bibliography”—a list of references or citations (9)	213	69
Identification of a primary source of health information: medical record (14)	195	63
Identification of references to journal articles from a list of references that includes both book references and article references (11)	187	61
Knowledge of a peer-reviewed journal article as an authoritative source of specialized health information (12)	185	60
Identification of a primary source of health information: hospital annual report (14)	173	56
Demonstration of a skill in locating a book in a university library catalogue based on a non-unique authors’ name and a general topic (15)	111	36
Knowledge of Boolean operators (*and, not, or*) (3)	105	34
Demonstration of a skill in setting up and performing a search with Boolean operators (*and, not, or*) (13)	98	32
**Evaluation of Information: Full-Text Journal Articles**		
Evaluation of journal articles: Identification of an article published prior to year 2000 (22)	248	80
Evaluation of journal articles: Identification of an article based on opinion rather than well-supported evidence (19)	242	79
Evaluation of journal articles: Identification of an article based on a review of existing research (20)	166	54
Evaluation of journal articles: Identification of an article written by an author whose affiliation is unknown (21)	148	48
**Evaluation of Information: Websites on Nutritional Supplements**		
Evidence-based decision-making: Disagree that “all three websites make a good case for taking nutritional supplements” (25)	187	61
Evaluation of health-related websites: Identification of the most trustworthy website (23)	154	50
Evaluation of health-related websites: Ability to identify the purpose of a website—to sell services (24)	42*	46
Evidence-based decision-making: Agree that “none of the websites makes a good case for taking nutritional supplements” (25)	67	22

^*^This question was added later, and, therefore, it had a smaller number of respondents (n = 92).

Note: RRSA question numbers are shown in parentheses; see Multimedia Appendix 1 for exact question wording.

To facilitate comparison of the three websites, we built in standard features that provided clues about high or low credibility. The standard features are URLs (two websites were .org and one was .com), links to the authors’ biographies, dates of publication, references, disclaimers, and links to organizations with which the authors are affiliated. These features act as contextual clues that maximize or minimize the trustworthiness of the websites. A review of such features is part of many website evaluation recommendations (for example, in their 1999 publication, Kotecki and Chamness [11] draw evaluators’ attention to a website’s features rather than its text), yet it is unclear if health information consumers are able to compare these features across multiple websites.

These standard features, rather than the text content, are intended to differentiate the websites in terms of their credibility. Because all respondents are equally uninformed about the nutritional supplements described in the text, they must attend to other features when making quality-related judgments. This purposeful design was motivated by the desire to avoid the confounding influence of pre-existing knowledge about the subject matter described in the document that is being judged. A good measure of one’s ability to critically evaluate Web pages is being able to disentangle the judgment of a website’s features from the judgment of its content. Study participants may have had preconceived notions about the quality of nutritional supplements depending on their purpose (e.g., cognitogenics are for sleeping disorders and gustatogenics are for appetite suppression). To avoid a possible interaction between the untrustworthy features of a website and the believable description of the nutritional supplement, we asked a group of students (n = 52) to judge the trustworthiness of the supplements’ descriptions presented as Microsoft Word documents rather than as websites. Although the level of trustworthiness was about the same for all nutritional supplement descriptions, the least trusted nutritional supplements were placed on the website with the highest number of untrustworthy features.

When five subject matter experts independently reviewed the three websites and rated their trustworthiness using the Kotecki and Chamness [11] website evaluation tool, they reached 100% agreement regarding the most trustworthy site. In comparison, undergraduates’ performance was much poorer: only 50% of respondents were able to identify the most trustworthy website (see Table 1).

### Understanding the Difference Between Scholarly Resources and Sales Pitches

Less than half of respondents determined the purpose of the least trustworthy website, which was to sell products and services. The visitors to this .com website are charged for reprints of the content, offered discounted products, and provided with multiple prompts (e.g., a running line) to book a consulting appointment with a private nutritionist who has few relevant qualifications. Customer testimonials posted on this site describe fantastic outcomes achieved within an unrealistically short time frame.

Less than a quarter of study participants reached the correct conclusion that none of the websites made a good case for taking the nutritional supplements, whereas 39% of respondents thought that all three websites made a good case for taking the supplements.

### Understanding Plagiarism

Health care professionals are expected to share health information with others, for example, by summarizing information from a variety of sources and distributing it to patients and clients. Higher education programs prepare students to apply standard rules for acknowledging contributions by others and referencing idea sources. Because this skill set is expected to become an integral part of their professional ethics, we built the RRSA to include measures of students’ knowledge of plagiarism, their ability to recognize it, and their awareness of its penalties. Our results indicate that the vast majority of students (92%) know that their university may impose a severe penalty for plagiarism, up to and including expulsion. Table 2 and Table 3 display responses to sample questions that measure declarative knowledge of plagiarism. They show that many students are aware that common knowledge can be reproduced without references, whereas words written by others should be enclosed in quotation marks and accompanied by a complete reference. But when presented with more ambiguous examples of plagiarism, some study participants demonstrated misconceptions about what constitutes plagiarism. A surprisingly large number of respondents believed that it is appropriate to present another person’s ideas as their own without citing a specific source, especially if this person is a relative or if the original words have been slightly modified.

**Table 2:** Understanding plagiarism: when references are needed (n = 308)

**Which of the following can be reproduced without proper reference? Check all that apply:**	**Respondents With Correct Positive or Negative Answers**	
	**n**	**%**
Common knowledge^*^	294	96
Hospital board member’s point of view	264	86
My classmate’s ideas	232	75
Unpublished works	223	73
Spoken word	209	68
My dad’s political opinions	156	51

^*^Common knowledge can be reproduced without proper reference.

Note: Items are scored as +1 if the answer is a correct positive or a correct negative and +0 if the answer is a false positive or a false negative.

**Table 3:** Defining plagiarism (n = 308)

**Which of the following are plagiarism examples? Check all that apply:**	**Respondents With Correct Positive or Negative Answers**	
	**n**	**%**
Submitting a free research paper that was downloaded off the Internet.^*^	290	95
Reproducing a sentence that you found quoted in a book without referring to the original source.^*^	276	90
Enclosing the word-for-word sentence in quotation marks, accompanied by a citation.	271	88
Copying from the source verbatim without any quotation marks but adding a citation.^*^	215	70
Putting someone’s idea in my own words without citing a specific source.^*^	201	65
Using similar sentence structure to express another person’s ideas without referring to the original source.^*^	169	55

^*^These items are examples of plagiarism..

Note: Items are scored as +1 if the answer is a correct positive or a correct negative and +0 if the answer is a false positive or a false negative.

To measure procedural knowledge of plagiarism, we ask respondents to compare a sentence from a *Health Affairs* article by Lapetina and Armstrong [33] to two other sentences that may have been plagiarized (question 20). Over two thirds of respondents (82%, n = 253) detected plagiarism in a sentence that closely follows the original but provides no reference to the original source. The percent of respondents who correctly identified a sentence without plagiarism (89%, n = 275) was comparable to the percent of respondents who knew that they should enclose the word-for-word sentence in quotation marks and cite the source (88%, n = 271, as shown in Table 3).

### Awareness of Personal Health Information Competencies

When asked “How do you rate your research skills overall?” most respondents (84%) believed that their skills were good, very good, or excellent. To compare self-reported and actual skill levels, we computed an overall health information competency score for each participant. An acceptable level of internal consistency reliability (Cronbach alpha = .78) for 56 right/wrong items indicates that it is appropriate to calculate the overall score as the sum of points of these 56 items. The overall scores ranged from 20 to 54 with a mean of 37 (SD = 6.35) and did not significantly depart from a normal distribution.

Actual performance was examined by self-reported skill level. The group differences were mostly in the expected direction (see Table 4), but there was a large amount of variation in the overall score *within* each self-reported skill level. This indicates that the overall health information competency score was high for some students and low for other students, despite the fact that their self-reported competency was the same.

**Table 4:** Means for health information competency overall score by self-reported skill level

**How do you rate your research skills?**	**n**	**Mean Overall Score**	**SD**
Nonexistent	0	-	0
Poor	3	36.33	4.04
Fair	47	34.89	5.52
Good	162	36.89	6.29
Very good	83	37.64	6.89
Excellent	13	36.77	6.10
Total	308	36.78	6.35

Health information competencies may vary as a function of education; therefore, we regressed undergraduates’ overall scores on the amount of credit hours earned toward the bachelor’s degree (Step 1) and self-reported skill level (Step 2). The level of education was operationalized as the number of credit hours earned (0-9, 10-24, 25-40, 41-70, and more than 71). The analysis was conducted for 302 undergraduate students (six graduate students were removed from this analysis). Age could not be used as a control variable because most students (95%) fell into the same category of 18 to 23 years of age. The variables entered on Steps 1 and 2 account for 8% of variance in the overall score (*R^2^* = .08). The amount of education significantly predicted the overall score (β = .28, *P <* .001). When credit hours earned were held constant, self-reports of skill fail to explain a significant amount of variance in the overall score (β = .08, *P* = .23). Overall, the results suggest that although students’ self-ratings of research skills tend to increase with the increasing level of education, these self-reports may not be an accurate predictor of students’ actual health information competencies.

## Discussion

### Interpretation of Findings

The present study represents a systematic effort to measure health information competencies using a standardized and reliable measurement tool, the Research Readiness Self-Assessment (RRSA). The data were obtained from a diverse sample of 308 respondents (77% response rate). Nonrespondents (n = 92) differed from respondents (n = 308) in terms of their academic level: freshmen were slightly more likely not to participate in the RRSA than higher-level students. The most likely explanation for nonparticipation is a lack of interest in extra credit rather than the computer-assisted administration of the RRSA. It is possible, of course, that students with particularly poor computer skills found the online administration a barrier. However, a semester after we collected the data reported in this paper, there was a 100% participation rate by 180 undergraduates in two introductory courses where the instructors required RRSA completion. The two course instructors reported no student complaints about not being able to follow emailed instructions on how to complete the assessment.

The data indicate that many students lack important competencies that may limit their ability to make informed health choices. We observed deficiencies in the areas of conducting advanced searches, discriminating among different types of information sources, referencing other people’s ideas, and evaluating information from Web pages and journal articles. Our data suggest that undergraduate students are inaccurate judges of their own competencies and hold a very positive view of their ability to do research. This finding may reveal an important barrier to building health information competencies of college-age students.

We found that there is a large competency gap between the average and the best information consumer. An average undergraduate in our sample is able to solve only 68% of problems that are solved by the best performing study participant (an average score of 37 versus a maximum score of 54). Health information competencies are applied to transform health-related information into knowledge that is consistent with the most current medical practice. High competence variability is a proxy indicator of students’ varying ability to make evidence-based decisions. In the past, limited access to information may have prevented health information consumers from acquiring knowledge and making informed choices. The new generation of health information consumers has, for the most part, easy access to information; yet it may not be able to take full advantage of this convenient access.

Our study shows that individuals with limited health information competencies may fail to locate the best available information due to employing poor search strategies. Searches that do not take into account all of the important criteria often produce low-relevancy documents or documents from commercial websites that promote products or services. These sites often present one-sided evidence, which can be detrimental to making a good decision about one’s health. Overall, many students are rather unsophisticated information consumers who rely on basic searchers and the easiest ways of retrieving information.

We found that many individuals know little about information sources—primary versus secondary, articles versus books, commercial versus noncommercial websites, and opinion pieces versus empirical studies. Information consumers who do not understand these distinctions are likely to engage in information processing that is shallow and superficial. They may, for example, follow a search path that produces the highest number of documents, rather than a path that produces documents of the highest quality. When the number of documents criterion is applied, Google and Yahoo significantly outperform all scholarly databases available through libraries. For instance, a Google search for the keyword *health* produces, in less than a second, over 8 million results ordered by popularity (as of June 2005, 25% of these results had .com URLs and 16% had .org or .gov URLs), where a similar search in Medline Plus produces 665 results, organized by health topic. With heavy reliance on public-access Internet search engines, an Information Age generation student may have an inaccurate conception that the Internet is the only place where society stores its best knowledge.

Once the plethora of documents is obtained, they need to be critically evaluated. Although health consumers are warned to critically examine websites to determine the document’s purpose, author’s affiliation, date of publication, and other features [11-14], these website evaluation criteria are only useful to those who know how to apply them. Many students in our sample appear not to possess these skills, and this finding is consistent with other observational studies (e.g., [17]). Our website evaluation exercise reveals both poor judgment and readiness to follow the lead, even when the authors of the online documents do not explicitly ask for purchase of their products. Although we measured a behavioral intent, rather than an actual behavior, there is still a significant potential for harm, ranging from financial losses to negative health effects, if only a few individuals execute their intent to take nutritional supplements that can be best described as “fake” or “bogus.” As we designed the most trustworthy website for the RRSA, it was alarming to witness the ease of misrepresenting or even falsifying health information. In designing the trustworthy site, we tried to meet as many website evaluation criteria as possible, and it became very apparent that these criteria do not guarantee information accuracy. Even completely false information about nonexistent food supplements can be made to appear trustworthy, as though it comes from an authoritative source.

Indeed, there is no substitute for good judgment when it comes to navigating information. Because this good judgment is a product of both critical thinking and extensive knowledge of the subject matter being researched, we believe that higher education programs are uniquely positioned to develop health information competencies. However, initial work on developing Information Age competencies needs to be done at the K-12 level when children are beginning to be exposed to various sources of information, including the Internet.

In this study, we reviewed three broad categories of information competencies—obtaining information, evaluating information, and using information. Using information includes such behaviors as reaching evidence-based conclusions and sharing information with others, a behavior guided by one’s understanding of plagiarism. One study of plagiarism revealed that cyberplagiarism, or inappropriate use of phrases and ideas published on the Internet, is prevalent even among scholars [34]. Our findings suggest that, in college students, the plagiarism behavior may originate not only from motivation to cut corners (e.g., to cut-and-paste text without citations) but also from the lack of nuanced knowledge about plagiarism. The information revolution has rapidly intensified the exchange of ideas, but the distinction between plagiarism and proper acknowledgment of others’ ideas continues to be poorly understood. Many students, for example, think that they do not need to provide references for paraphrased sentences or for sentences whose structure they modified. Perhaps these students view plagiarism as a violation of ownership of *exact words* rather than a violation of ownership of *ideas*. Similarly, some respondents believe that it is appropriate not to give credit for original ideas that are expressed orally (rather than in writing) or by people whom they know well. If carried into one’s professional life, this misconception can make it difficult to follow ethical norms for recognizing others’ knowledge contributions. Such ethical norms are strong in health professions, and their violation may lead to negative consequences.

Perhaps the most interesting finding is the fact that participants are so unaware of their own skill deficiencies. It is possible that students make global judgments about their research skills based primarily on their ability to access information. That is, one’s ability to access information may be confused with one’s ability to generate knowledge from the information accessed. But obtaining information is merely the first step of knowledge acquisition. All of our study participants can access the Internet, as demonstrated by completing the RRSA online, but not all may be able to make good use of the information they access. Extending the argument by Solomon and Leigh [9] from television to Internet search engines, we conclude that the effort an individual expends to locate millions of documents in Google is a poor indicator of the true effort needed to process the obtained material “if one aimed at a deeper understanding of it” [9]. The Information Age generation of college students may benefit from this point.

### Implications for Health Promotion Practice

The findings of our study have several implications for individuals who practice health promotion for health information consumers. Health educators, librarians, and other professionals who play an active part in promoting health information literacy need to assist health information consumers in becoming more aware of their skill limitations. These professionals should develop their own proficiency in managing modern media and be able to find, evaluate, interpret, and present health-related information to other information consumers. Research on health information competencies of practicing health professionals remains limited, and we do not yet have a complete picture of their preparedness for evidence-based practice. But in one survey study of 1097 registered nurses, it was found that many respondents “had no exposure to the research process in their educational programs, do not appreciate the importance of research to practice, and have great difficulty understanding research articles” [35]. In this study, most registered nurses did not search databases such as Medline or felt skilled to do so. This preliminary evidence suggests that health professionals need to build their health information competencies.

The RRSA instrument offers an operational definition of information literacy, which remains an ill-defined concept. Upon examination of 97 Medline articles on the topic of information literacy for health care professionals, Saranto and Hovenga [36] found that the concept of information literacy has not yet been established. It is sometimes used interchangeably with *computer literacy* and *informatics awareness* or with the ambiguous term *computer experience*. The RRSA assessment used in the present study adds to the literature on health literacy by defining basic knowledge and skills needed for managing electronic health information resources.

Among the limitations of the present study is the narrowly focused sample, which limits our ability to generalize the study’s findings to the broader population of health information consumers. The students from a Midwestern university may not be completely representative of the entire population of US Information Age students, due to, for example, the relatively homogeneous ethnic composition and possible overrepresentation of individuals raised in rural communities. In our future studies, we intend to broaden the pool of RRSA participants by including multiple educational institutions as well as urban and rural communities located in different geographic regions.

In contrast with many health information literacy studies, this research presents the results obtained via direct measure of skills and knowledge rather than via self-reports by health information consumers. While the reliability of the RRSA assessment reaches acceptable levels, it is necessary to further assess its unidimensionality, content validity, and criterion-related validity. A comprehensive validation study of the RRSA instrument is currently under way.

### Conclusions

Today, health consumers are actively seeking information and using it to make health decisions. The ease of accessing information may influence their perceptions of their ability to make informed health decisions. Our study shows that to become savvy information consumers, young people may need assistance in understanding the various health media, building awareness of their own skill sets, and improving their ability to make evidence-based decisions. Individuals with less education and exposure to information-related activities are expected to have even lower health information competencies than our study participants [37]. Health educators must continue to partner with a variety of groups that play an important role in promoting health information literacy, such as librarians and educators.

The assessment tool used in the present study is a self-administered instrument that provides a reliable account of health information competencies related to managing electronic health information. Data acquired through this research can be used to suggest curriculum improvements and estimates of the higher end level of skill held by health information consumers. It can also be used to educate health information consumers about their levels of skill necessary for managing health information from electronic sources. RRSA findings suggest that health information competencies of undergraduate students, many of whom will soon enter a variety of health professions, are limited. Health literacy educators can utilize RRSA findings to design educational interventions that impact information consumers’ skills and prepare them for the challenges of living and working in the Information Age.

## Multimedia Appendix 1

RRSA questions.

## Multimedia Appendix 2

Video of the online-administered RRSA instrument.

## Multimedia Appendix 3

Powerpoint slides about the RRSA study.

## Abbreviations

RRSA: Research Readiness Self-Assessment
