# Supplementary material for: Health Information Literacy and Competencies of Information Age Students: Results From the Interactive Online Research Readiness Self-Assessment (RRSA)
Source: J Med Internet Res. 2006 Apr 21;8(2):e6. doi: 10.2196/jmir.8.2.e6 (PMC1550696; doi:10.2196/jmir.8.2.e6)
Supplement: Supplementary file 1 [file jmir_v8i2e6_app1.doc]

**Appendix A**

**Questions Included in the Health Professions Version of**

**Research Readiness Self-Assessment (RRSA), May 2005**

**Note**. Listed below are select RRSA questions (survey items and test items) used in May 2005 to obtain the data discussed in the JMIR publication by Ivanitskaya, O’Boyle and Casey (2006). Some items contain inactive hyperlinks. The content of these hyperlinks is shown in an accompanying file titled *Video of RRSA.exe* (Appendix B). The RRSA instrument has been revised and improved since it was administered in May 2005. For the most up-to-date copy of the instrument, contact Lana Ivanitskaya, Ph.D. at [ivani1sv@cmich.edu](mailto:ivani1sv@cmich.edu)

**1 - How do you rate your research skills overall?**

__ Excellent

__ Very good

__ Good

__ Fair

__ Poor

__ Nonexistent

**2 - Your instructor has just assigned a research paper on any topic related to health education. Which of the following are the best ways to start? Check all that apply:**

__ Just start typing and see where it goes.

__ Find everything I can on health education.

__ I'd think about the topic, the assignment and my particular interests then I'd decide what I'd like to write about.

__ Contact a library staff member for advice on common health education topics.

**3 - One can retrieve the most documents in an online library catalog by searching for:**

__ Health and wellness

__ Health not wellness

__ Health or wellness

__ Not health wellness

__ Or health and wellness

**4 - Searching for the keyword "health" in an online library catalog is most likely to provide...**

__ a fairly complete list of general resources about the United States Department of Health and Human Services

__ an overwhelmingly large number of resources on a variety of topics

__ links to detailed budget statements of major health organizations

__ an annotated bibliography of health sciences research

__ a list of documents by the World Health Organization

**5 - Which of the following are examples of plagiarism? Check all that apply:**

__ Using similar sentence structure to express another person's ideas

__ Enclosing the word-for-word sentence in quotation marks, accompanied by a citation

__ Reproducing information that appears in many sources and that is easily available without citing a specific source

__ Putting someone's idea in my own words without citing a specific source

__ Reproducing a sentence that you found quoted in a book without referring to the original source

__ Submitting a free research paper that was downloaded off the Internet

__ Copying from the source verbatim without any quotation marks but adding a citation

__ Making up a quotation that does not exist

**6 - What is the most authoritative source of current scholarly (analytical) information on a narrowly specialized topic?**

__ Web search engines, such as Google or Yahoo

__ Textbook

__ Newspaper

__ Book

__ Journal

**7 - Which of the following titles are scholarly or academic journals? Check all that apply:**

__ U.S. News and World Report

__ Health Services Research

__ Time

__ Journal of the American Medical Association

__ The Wall Street Journal

**8 - A journal article abstract is...**

__ A summary of other research on this topic

__ A summary of the article's content (e.g., purpose, methodology, findings and conclusions)

__ An annotated list of references used in the article

__ A note or paragraph about the authors of the article

__ A glossary of abstract concepts and constructs included in the researchers' model

**9 - A bibliography is:**

__ A book about a person's life

__ A book of charts and graphs

__ A list of references or citations

__ A directory of names

__ Bible geography, a historical perspective into where the Bible events occurred

**10 - Which of the following can be reproduced without a proper reference? Check all that apply:**

__ Hospital board member's point of view

__ Spoken word

__ Common knowledge

__ My classmate's ideas

__ Unpublished works

__ My dad's political opinions

**11 - The following citations are in APA Style, 5th edition. Which of these citations are to journal articles? Check all that apply:**

__ Hartley, D., Moscovice, I., & Christianson, J. (1996). Mobile technology in rural hospitals: The case of the CT scanner. Health Services Research, 31, 213-234.

__ Ganz, P. A. (2002). What outcomes matter to patients: A physician-researcher point of view. Medical Care, 40, 11-19.

__ Lowe, J. I., & Herranen, M. (1982). Understanding teamwork: Another look at concepts. Social Work in Health Care, 7(2), 1-11.

__ De Vellis, R. F. (1991). Scale Development: Theory and Applications. Newbury Park, CA: Sage.

__ Kohn, L. T., Corrigan, J. M., & Donaldson, M. S. (Eds.). (1999). To Err is Human: Building a Safer Health Care System. Washington, DC: National Academy Press.

__ Nezu, C. M., Tsang, S., & Lombardo, E. R. (2003). Complementary and alternative therapies, In Nezu, A. M., & Nezu, C. M. (Eds.). Handbook of Psychology: Health Psychology (vol. 9, pp. 591-614). New York: John Wiley & Sons.

**12 - When quoting information about the impact of inadequate nurse staffing on patient health, it is best to use:**

__ the most up-to-date source found on the Internet

__ a peer-reviewed journal article

__ a conversation overheard by an instructor

__ a quote from a newsletter

__ services provided by a very experienced lawyer

**13 - You are interested in gathering information about work stress, but are not interested in its medical side effects. Set up a document search in a separate window using the following keywords: “stress” “medical”**

**<hyperlink>Click here to begin your search**

**In the space provided, report the number of documents you found: _____________**

**14 - Which of the following materials are primary and not secondary sources of information? Check all that apply:**

__ A physiology textbook

__ A Mayo Clinic annual report

__ My medical record

__ A newspaper article about the number of deaths due to homicide

__ A summary of empirical studies that had the greatest impact on health policy

**15 - Your paper topic is "health information systems." You have misplaced the source you quoted in your paper:**

**Tan (2001) provided an in-depth overview of the systems used to store and retrieve health-related information.**

**Search Central Michigan University Libraries' Online Catalog at http://catalog.lib.cmich.edu/ to find this book's 10-digit ISBN number. Enter the ISBN number here: _____________**

**16 - You have found a good reference for your research paper. It is a book edited by Williams and others. The book title is “The epidemiology of diabetes mellitus: An international perspective.” Find it by searching the library catalog: http://catalog.lib.cmich.edu/ What is the CALL NUMBER of this book?**

__ OA 645.D5 E654

__ PA 645.D5 E654

__ RA 645.D5 E654

__ SA 645.D5 E654

__ TA 645.D5 E654

**17 - A complete citation is needed to obtain an article or a book. Which of the following citations lack important information? Check all that apply:**

__ LoGerfo, J. P., & Rubenstein, L. Z. (2003). Preventing falls in elderly persons. New England Journal of Medicine, 348, 1816-1818.

__ Improving public health through policy advocacy. Community-based Public Health Policy & Practice, 8, 1-8.

__ Wheeler, L. (2003). Asthma management in schools. FDA Consumer, 37(2), 40.

__ McNulty, T., & Ferlie, E. (2002). Reengineering health care: the complexities of organizational transformation.

__ Loue, S., Lloyd, L.S., & O’Shea, D. Community health advocacy. New York: Kluwer Academic/Plenum Publishers.

**18 - Below is a passage taken from an article by Lapetina and Armstrong (2002):**

**Some errors in cosmetic surgery could be prevented if patients were made aware of their physician's level of training.**

**Which of the following represent a plagiarized version of the above sentence? Check all that apply:**

__ If patients are told about their physician's level of training, there will be fewer errors in cosmetic surgery (Lapetina & Armstrong, 2002).

__ Given the elective nature of cosmetic surgery procedures and the frequency of post-operative complications, it has been recommended that patients should be "made aware of their physician's level of training" (Lapetina & Armstrong, 2002, p.36).

__ Because there are many medical errors in cosmetic surgery, patients should be made aware of their physician's level of training.

__ Some errors in cosmetic surgery might be averted through consumers' awareness of a physician's level of education (Lapetina & Armstrong, 2002).

**19 - You need to find 3 research articles on job satisfaction. You used the best resource available to you - the password-protected databases that are accessible through your library - and found these three full-text articles:**

**<hyperlink>Improving professional satisfaction**

**<hyperlink>The joy of work**

**<hyperlink>The effects of employee satisfaction**

**Click on the links to examine each article and evaluate its content.**

**Which of these articles is based on opinion, rather than well-supported evidence?**

__ Improving professional satisfaction

__ The joy of work

__ The effects of employee satisfaction

**20 - Which article is based on a review of existing research on job satisfaction?**

__ Improving professional satisfaction

__ The joy of work

__ The effects of employee satisfaction

**21 - Which article was written by an author whose affiliation is unknown?**

__ Improving professional satisfaction

__ The joy of work

__ The effects of employee satisfaction

**22 - Which article was published prior to year 2000?**

__ Improving professional satisfaction

__ Joy of work

__ The effects of employee satisfaction

**23 - You are looking for information on various nutritional supplements. You found three web sites:**

**<hyperlink>Cognitogenic aids**

**<hyperlink>Dormitogenic aids**

**<hyperlink>Vescorogenic (gustatogenic) aids**

**Click on the links to examine each web site and evaluate its content. Which of these web sites is the most trustworthy?**

__ Cognitogenic aids

__ Dormitogenic aids

__ Vescorogenic (gustatogenic) aids

**24 - What is the most likely purpose of the vescorogenic (gustatogenic) aids web site?**

__ To assist a large number of people in achieving their nutrition and wellness goals

__ To educate people that FDA did not evaluate the effectiveness of vescorogenic (gustatogenic) aids

__ To promote research on how vescorogenic (gustatogenic) aids inhibit body fat biosynthesis

__ To sell John Goode’s services and vescorogenic (gustatogenic) aids

__ To explain that not all "diet drugs" are vescorogenic (gustatogenic) aids

__ To provide rich, evidence-based information on how vescorogenic (gustatogenic) aids helped specific people get healthy

**25 - Check statements that are true about the three web sites you evaluated:**

**<hyperlink>Cognitogenic aids**

**<hyperlink>Dormitogenic aids**

**<hyperlink>Vescorogenic (gustatogenic) aids**

**Check all that apply:**

__ I may recommend that people suffering from learning disabilities take supplements described in cognitogenic aids

__ I may recommend that people suffering from sleeping disorders take supplements described in dormitogenic aids

__ I may recommend that people suffering from obesity take supplements described in vescorogenic (gustatogenic) aids

__ All three web sites make a good case for taking nutritional supplements

__ None of the web sites make a good case for taking nutritional supplements

**26 - Your current academic status:**

__ Undergraduate

__ Graduate

__ Non-degree

__ Other

**27 - How many credits have you earned toward your next degree?**

__ none

__ 1-9

__ 10-24

__ 25-40

__ 41-70

__ 71+

**29 – Your Gender:**

__ Male

__ Female

**30 - Your age:**

__ Under 18

__ 18-23

__ 24-30

__ 31-40

__ 41-50

__ 51-60

__ 60+
