# Supplementary material for: Health Information Literacy and Competencies of Information Age Students: Results From the Interactive Online Research Readiness Self-Assessment (RRSA)
Source: J Med Internet Res. 2006 Apr 21;8(2):e6. doi: 10.2196/jmir.8.2.e6 (PMC1550696; doi:10.2196/jmir.8.2.e6)
Supplement: Supplementary file 3 [file jmir_v8i2e6_app2b.html]

RRSA-JMIR
