# Supplementary material for: Health Information Literacy and Competencies of Information Age Students: Results From the Interactive Online Research Readiness Self-Assessment (RRSA)
Source: J Med Internet Res. 2006 Apr 21;8(2):e6. doi: 10.2196/jmir.8.2.e6 (PMC1550696; doi:10.2196/jmir.8.2.e6)
Supplement: Supplementary file 4 [file jmir_v8i2e6_app3.ppt]

## Slide 1
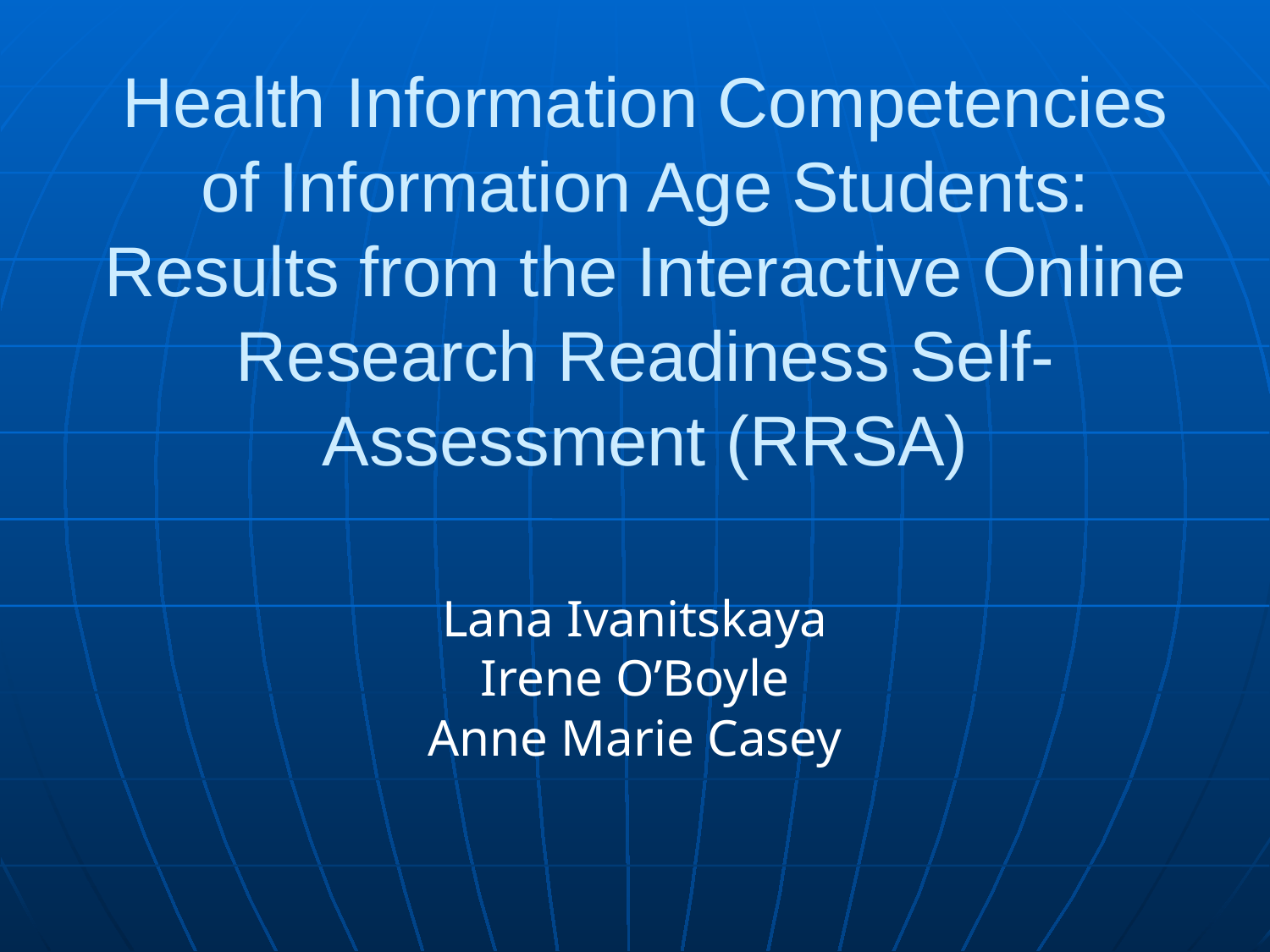

# Health Information Competencies of Information Age Students: Results from the Interactive Online Research Readiness Self-Assessment (RRSA)
Lana Ivanitskaya
Irene O’Boyle
Anne Marie Casey

## Slide 2
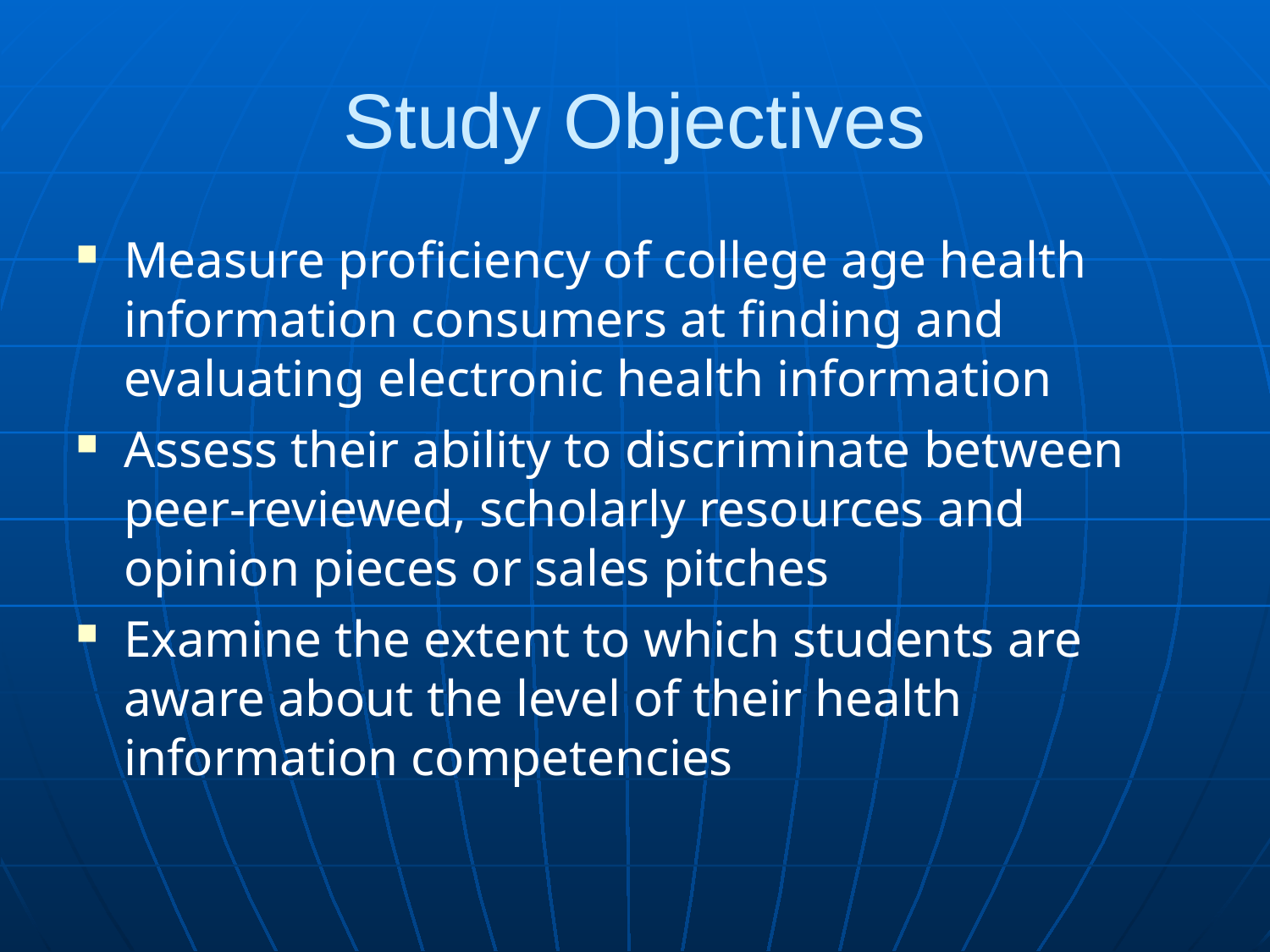

# Study Objectives
Measure proficiency of college age health information consumers at finding and evaluating electronic health information
Assess their ability to discriminate between peer-reviewed, scholarly resources and opinion pieces or sales pitches
Examine the extent to which students are aware about the level of their health information competencies

## Slide 3
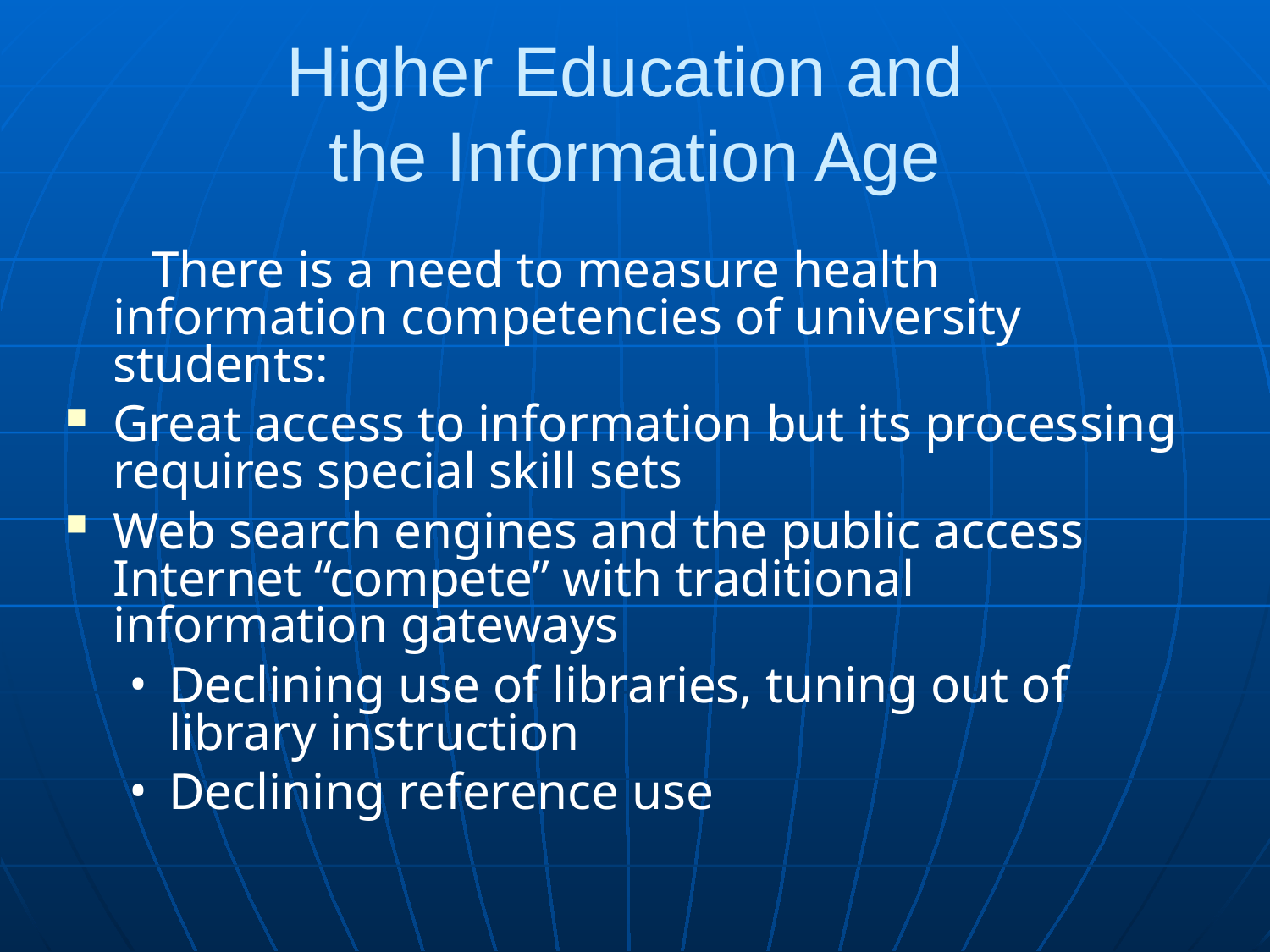

# Higher Education and the Information Age
 There is a need to measure health information competencies of university students:
Great access to information but its processing requires special skill sets
Web search engines and the public access Internet “compete” with traditional information gateways
Declining use of libraries, tuning out of library instruction
Declining reference use

## Slide 4
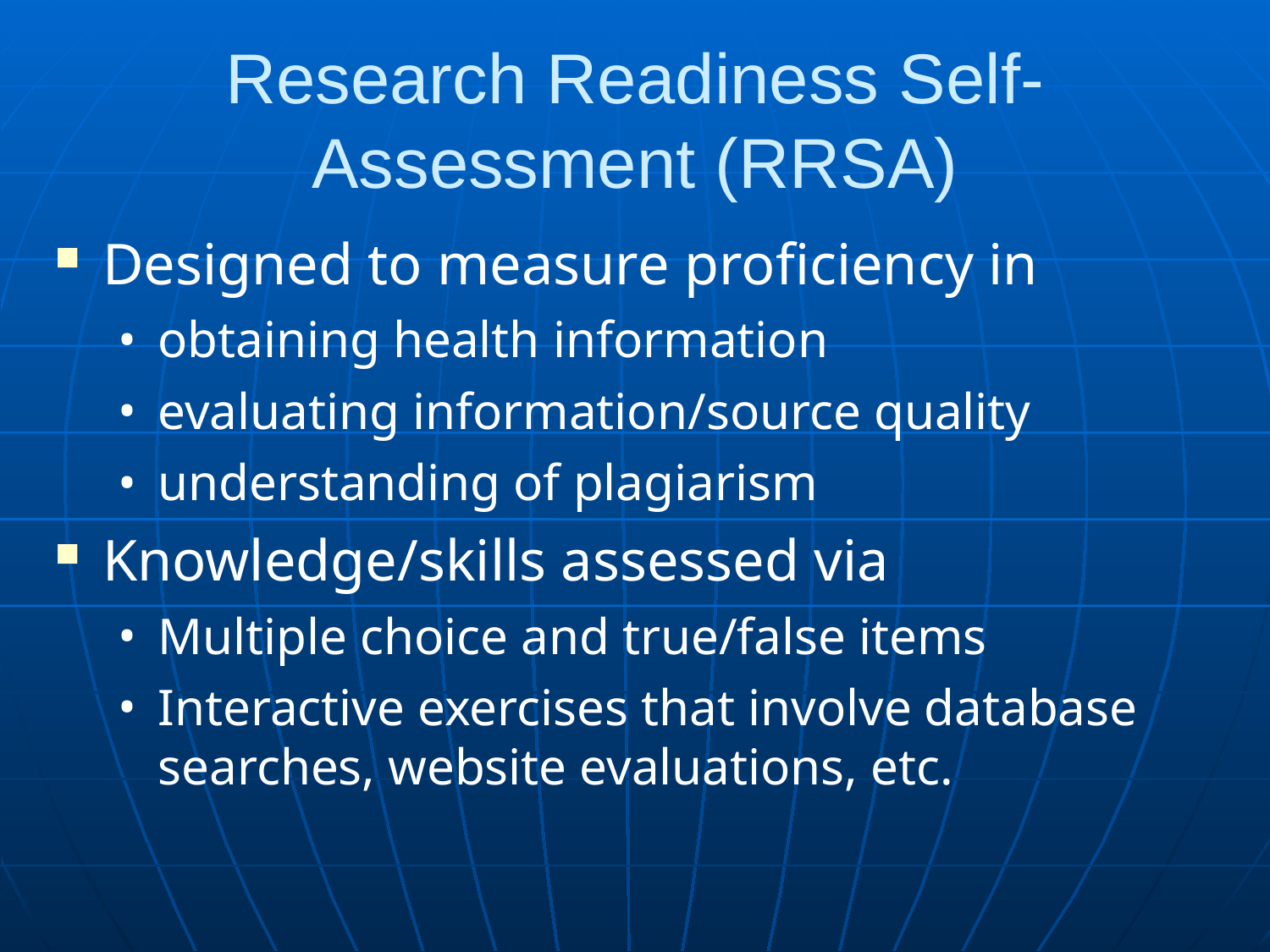

# Research Readiness Self-Assessment (RRSA)
Designed to measure proficiency in
obtaining health information
evaluating information/source quality
understanding of plagiarism
Knowledge/skills assessed via
Multiple choice and true/false items
Interactive exercises that involve database searches, website evaluations, etc.

## Slide 5
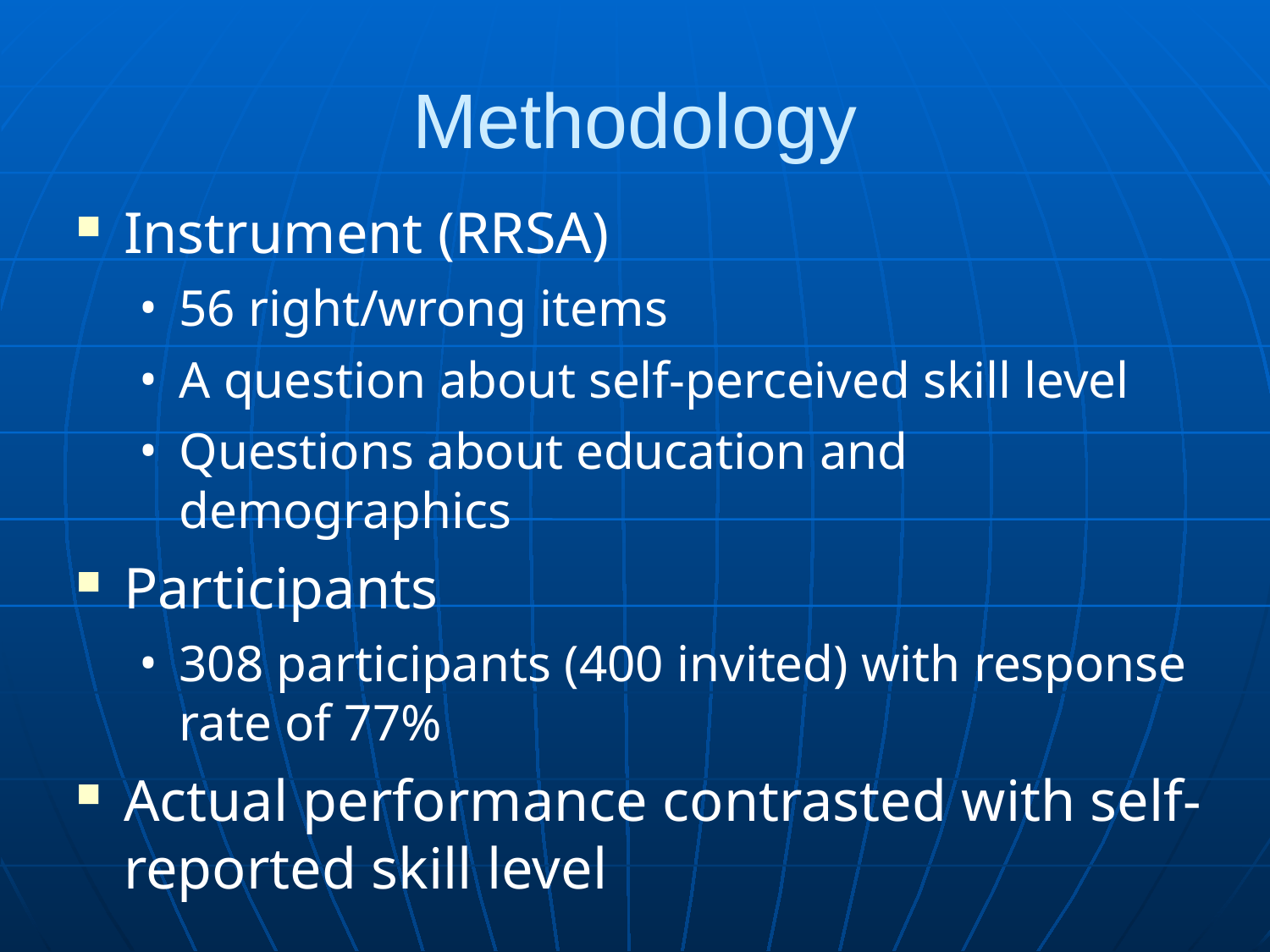

# Methodology
Instrument (RRSA)
56 right/wrong items
A question about self-perceived skill level
Questions about education and demographics
Participants
308 participants (400 invited) with response rate of 77%
Actual performance contrasted with self-reported skill level

## Slide 6
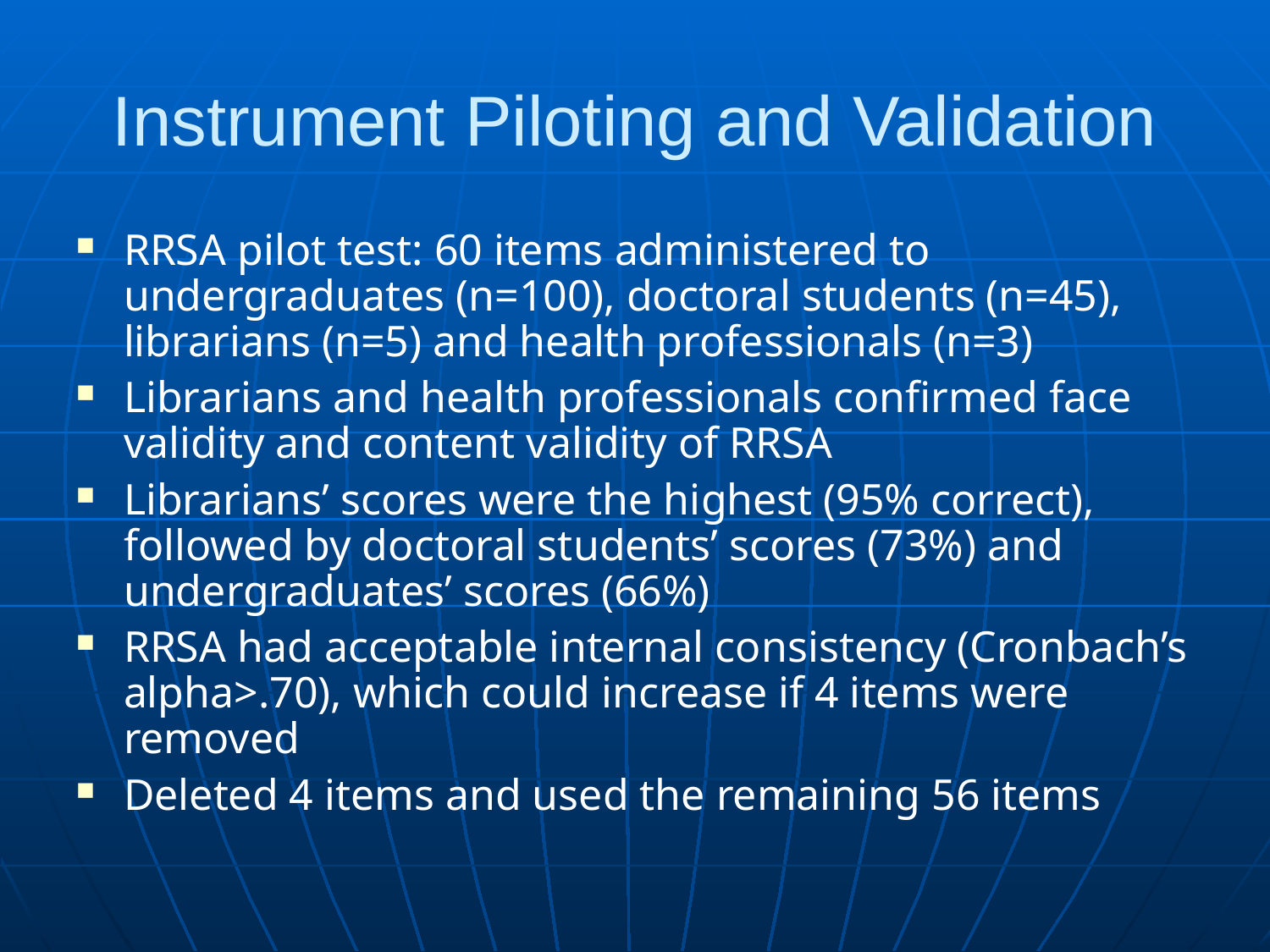

# Instrument Piloting and Validation
RRSA pilot test: 60 items administered to undergraduates (n=100), doctoral students (n=45), librarians (n=5) and health professionals (n=3)
Librarians and health professionals confirmed face validity and content validity of RRSA
Librarians’ scores were the highest (95% correct), followed by doctoral students’ scores (73%) and undergraduates’ scores (66%)
RRSA had acceptable internal consistency (Cronbach’s alpha>.70), which could increase if 4 items were removed
Deleted 4 items and used the remaining 56 items

## Slide 7
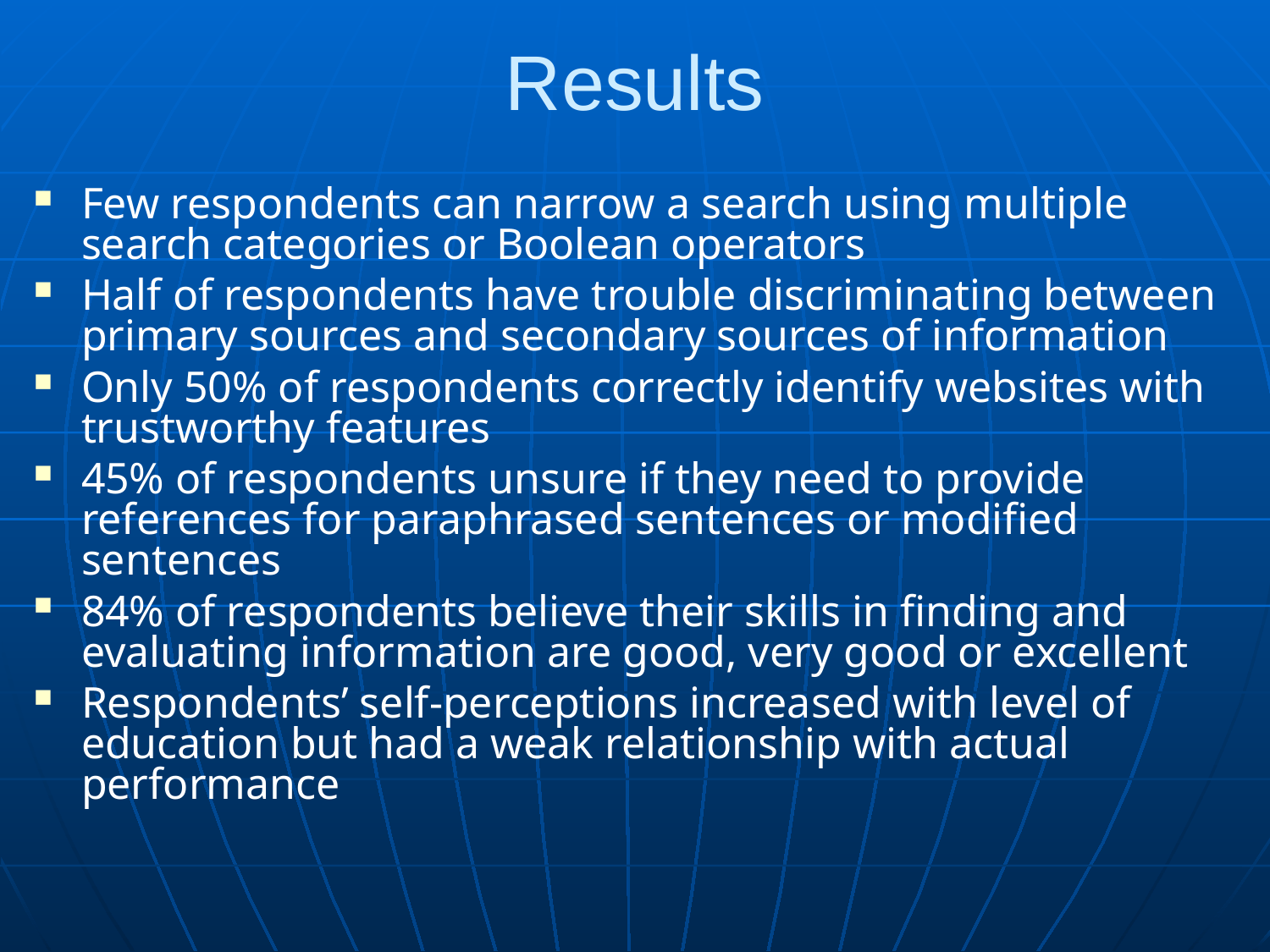

# Results
Few respondents can narrow a search using multiple search categories or Boolean operators
Half of respondents have trouble discriminating between primary sources and secondary sources of information
Only 50% of respondents correctly identify websites with trustworthy features
45% of respondents unsure if they need to provide references for paraphrased sentences or modified sentences
84% of respondents believe their skills in finding and evaluating information are good, very good or excellent
Respondents’ self-perceptions increased with level of education but had a weak relationship with actual performance

## Slide 8
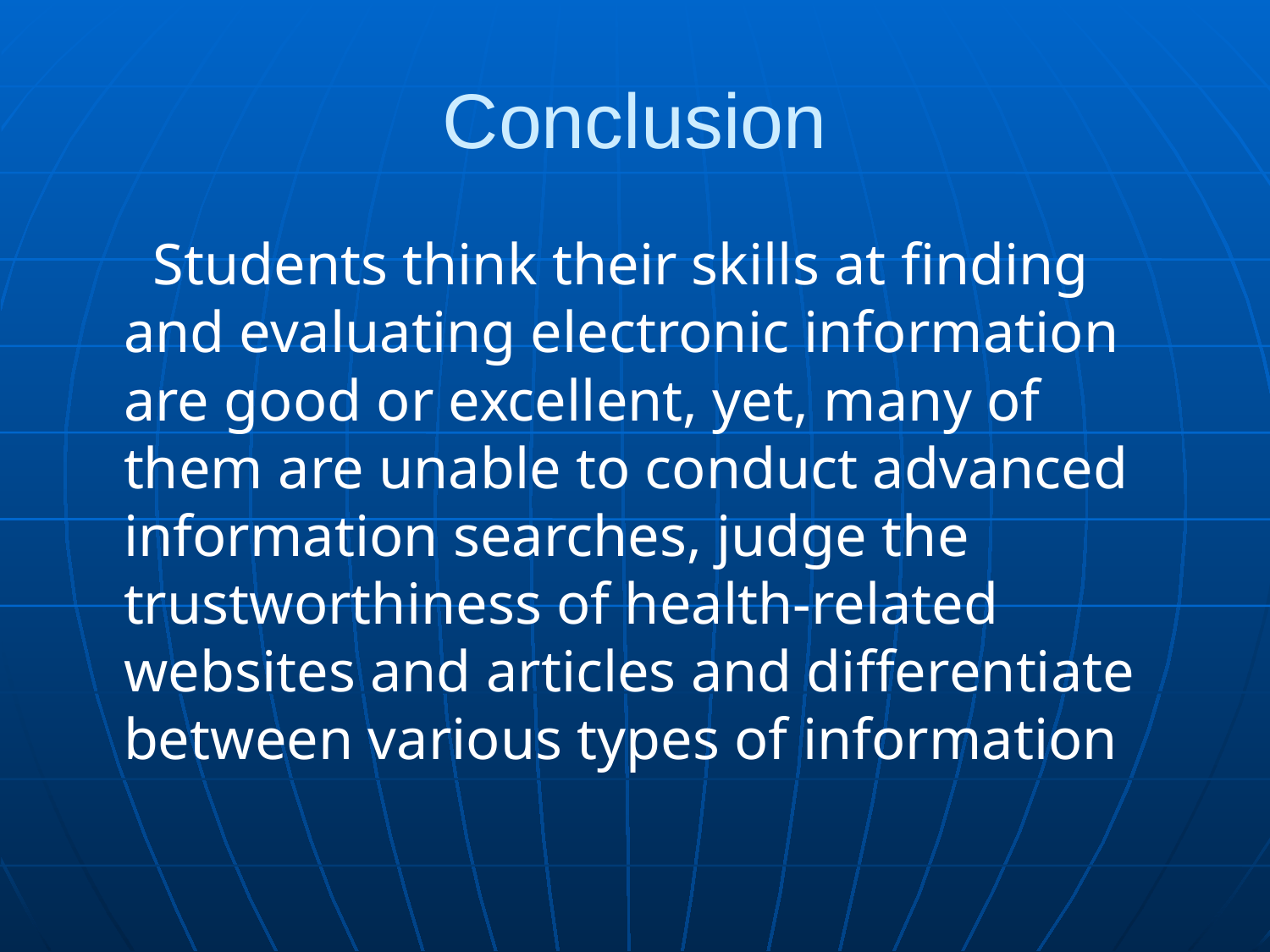

# Conclusion
 Students think their skills at finding and evaluating electronic information are good or excellent, yet, many of them are unable to conduct advanced information searches, judge the trustworthiness of health-related websites and articles and differentiate between various types of information

## Slide 9
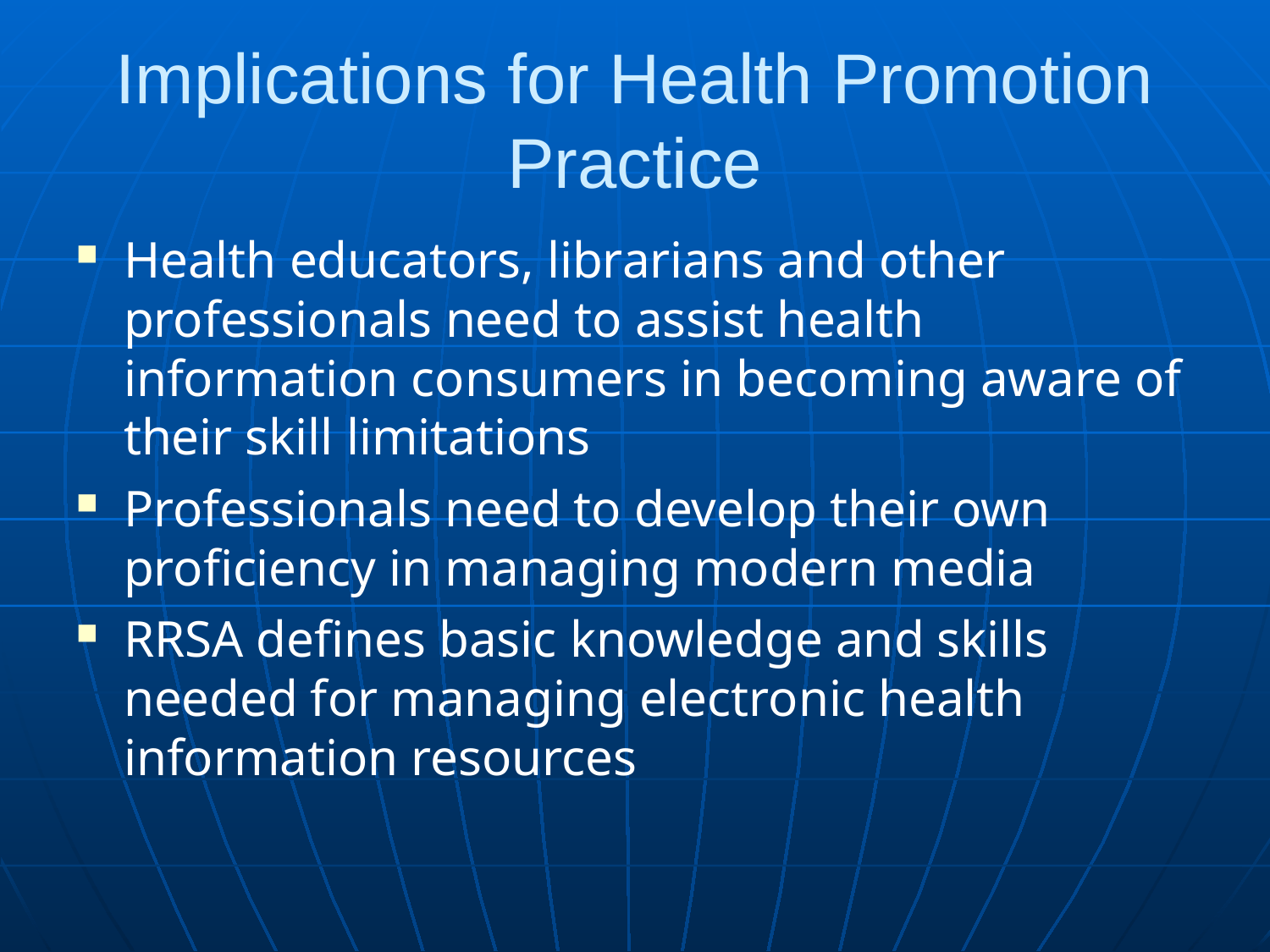

# Implications for Health Promotion Practice
Health educators, librarians and other professionals need to assist health information consumers in becoming aware of their skill limitations
Professionals need to develop their own proficiency in managing modern media
RRSA defines basic knowledge and skills needed for managing electronic health information resources

## Slide 10
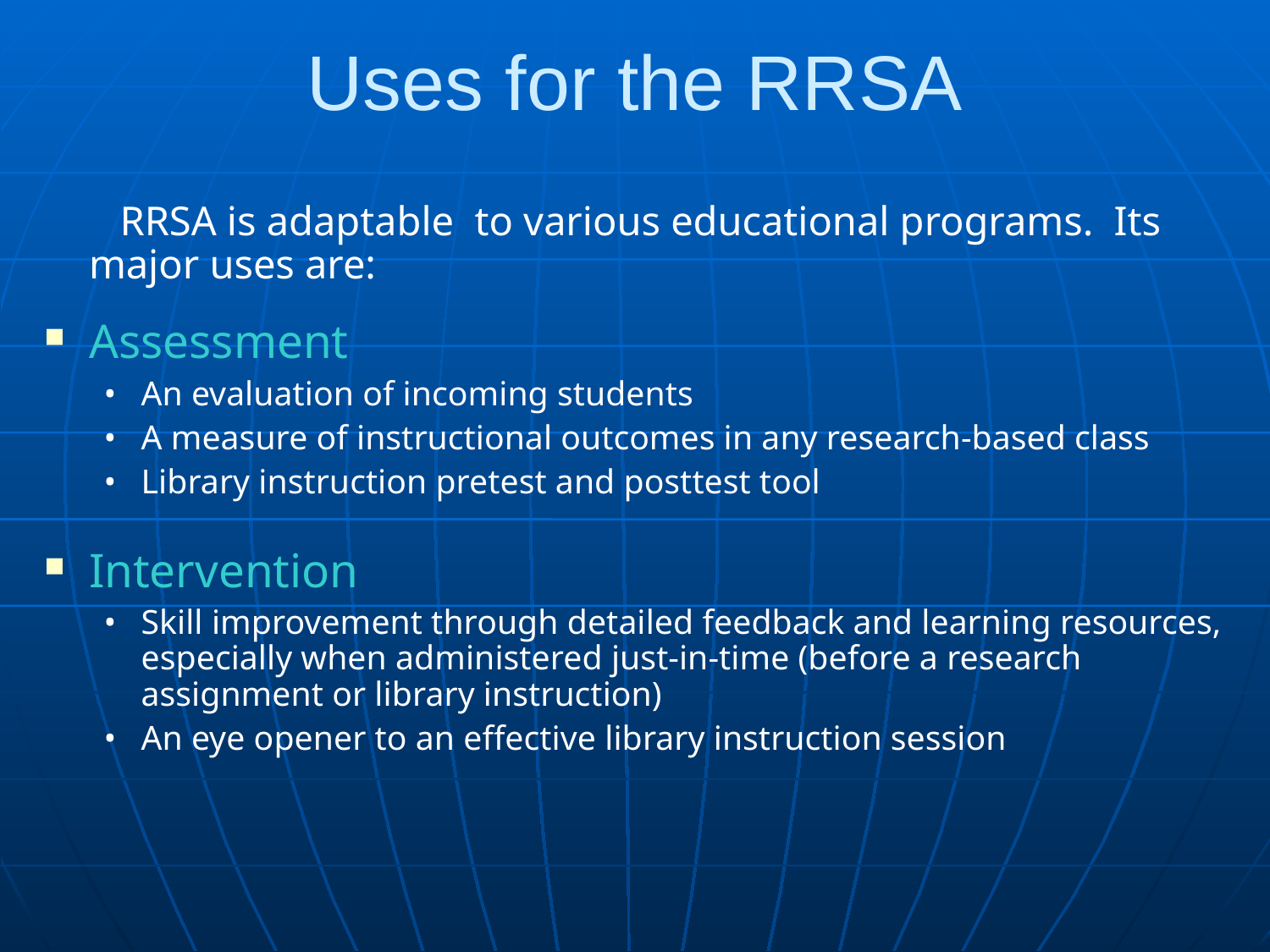

# Uses for the RRSA
 RRSA is adaptable to various educational programs. Its major uses are:
Assessment
An evaluation of incoming students
A measure of instructional outcomes in any research-based class
Library instruction pretest and posttest tool
Intervention
Skill improvement through detailed feedback and learning resources, especially when administered just-in-time (before a research assignment or library instruction)
An eye opener to an effective library instruction session
